# The mediating effect of DHA-mediated MPV improvement on coronary artery stenosis and its prognostic value

**DOI:** 10.3389/fcvm.2026.1809653

**Published:** 2026-07-03

**Authors:** Wenli Wang, Changguo Ou, Jianqiu Liang, Dongliang Liang, Jiangxiong Wen, Yue Xie, Baohua Liu, Yangguang Liu, Yuli Huang, Jiandi Wu

**Affiliations:** 1 Department of Cardiology, Foshan Second People's Hospital, The Affiliated Foshan Hospital of Guangdong Pharmaceutical University, Foshan, China; 2 School of First Clinical Medicine, Guangdong Medical University, Zhanjiang, China; 3 Department of Cardiology, The Eighth Affiliated Hospital of Southern Medical University, Foshan, China

**Keywords:** coronary artery disease, docosahexaenoic acid, eicosapentaenoic acid, gensini score, major adverse cardiovascular events, mean platelet volume, mediating effect

## Abstract

**Background:**

Cardiovascular disease (CVD) remains a leading global health threat. The high incidence of coronary artery disease (CAD) is closely associated with atherosclerotic progression, and thrombosis mediated by platelet activation is a key mechanism triggering acute coronary events and contributing to the high mortality of CAD. Omega-3 polyunsaturated fatty acids (*ω*-3 PUFA) exert cardiovascular protective effects through anti-inflammatory and antiplatelet aggregation effects. However, their mechanisms underlying the regulation of coronary stenosis severity in CAD via platelet morphological parameters (mean platelet volume, MPV) and their prognostic value remain unclear. This study aims to investigate the association between serum DHA, EPA, and MPV with the onset of CAD, the severity of coronary artery stenosis (Gensini score), and the prognosis of major adverse cardiovascular events (MACE), while validating the mediating effect of MPV.

**Methods:**

A total of 183 patients with coronary artery disease (CAD) and 189 controls were included. Baseline data were collected, and levels of eicosapentaenoic acid (EPA), docosahexaenoic acid (DHA) and arachidonic acid (AA) were measured by ultra-performance liquid chromatography-mass spectrometry. Logistic regression, Spearman correlation analysis, bootstrap mediation analysis, Kaplan–Meier survival analysis, and Cox proportional hazards regression were employed to explore the mechanisms and associations between omega-3 PUFAs, MPV, and CAD onset, coronary artery stenosis severity, and MACE.

**Results:**

Serum DHA levels were significantly lower in the CAD group than in controls (*P* < 0.001). Among CAD patients, DHA was significantly negatively correlated with MPV, platelet distribution width (PDW), and platelet large cell ratio (PLCR) (*r* = −0.169, −0.156, −0.186, all *P* < 0.05), while MPV, PDW, and PLCR were weakly positively correlated with Gensini scores (*r* = 0.170, 0.158, 0.165, all *P* < 0.05). Bootstrap mediation analysis confirmed that MPV showed a statistically significant mediating effect in the association between DHA and Gensini score, consistent with a mediation model (*P* = 0.034). Prognostic analyses demonstrated that DHA was an independent protective factor for MACE (HR = 0.83, 95% CI: 0.74–0.93, *P* = 0.002),MPV was an independent risk factor for MACE (HR = 1.22, 95% CI: 1.00–1.49, *P* = 0.046), and the combination of “high DHA (≥1.4127 μmol/L) + low MPV (≤10.4 fL)” significantly reduced the risk of MACE (Log-rank test, *P* = 0.0011).

**Conclusion:**

DHA is associated with coronary stenosis severity, and this association is statistically consistent with partial mediation by MPV. The combined assessment of DHA and MPV optimizes risk stratification in CAD patients, providing quantitative evidence for personalized nutritional interventions with significant clinical translational value.

## 1 Introduction

Cardiovascular disease (CVD) remains the leading cause of death globally, with coronary atherosclerotic disease (CAD) being its predominant form, imposing a significant global disease burden (1). Within the complex pathogenesis of CAD, platelet activation not only functions as a core driver of acute thrombosis but also directly contributes to and accelerates atherosclerotic progression by promoting inflammatory responses (2, 3). However, direct detection of platelet activity is associated with clinical limitations including operational complexity and high costs, making the identification of reliable and validated surrogate markers critical. Among various indicators, mean platelet volume (MPV) has emerged as a key and readily accessible biomarker reflecting platelet activation owing to its ease of acquisition and low cost. Accumulating evidence confirms that MPV is significantly associated with the severity of coronary artery lesions and the risk of major adverse cardiovascular events (MACE) (4–7).

**Figure 1:**
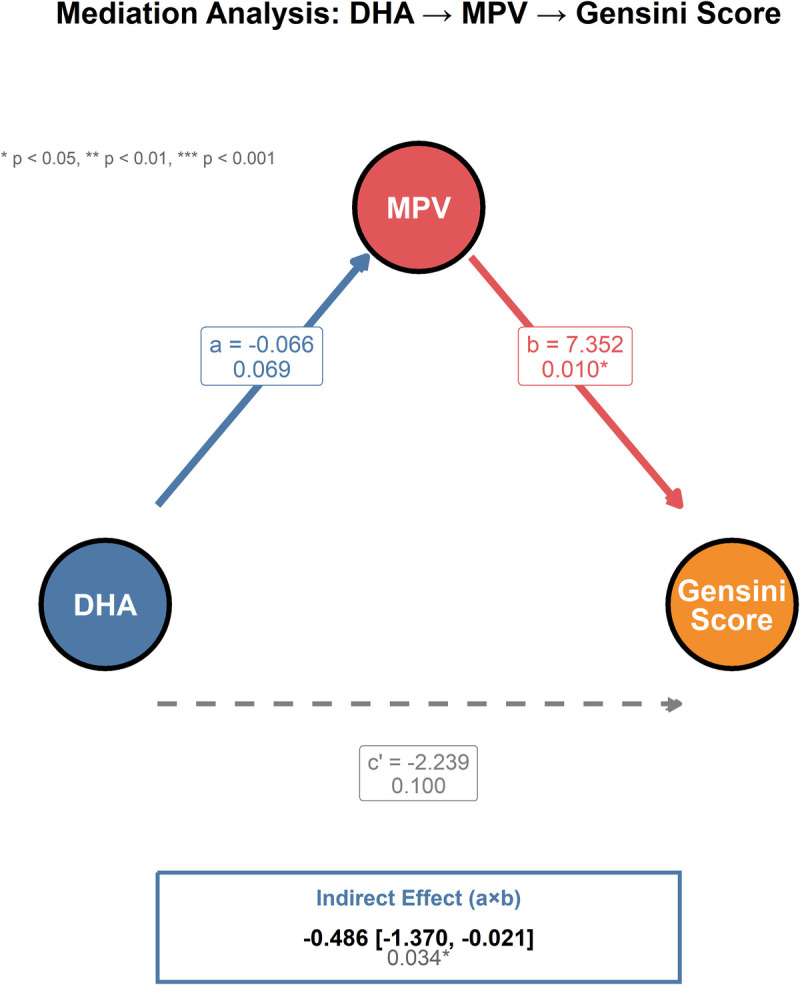
Analysis of the mediating effect of MPV on the relationship between DHA and gensini score. DHA, docosahexaenoic acid; MPV, mean platelet volume.

**Figure 2:**
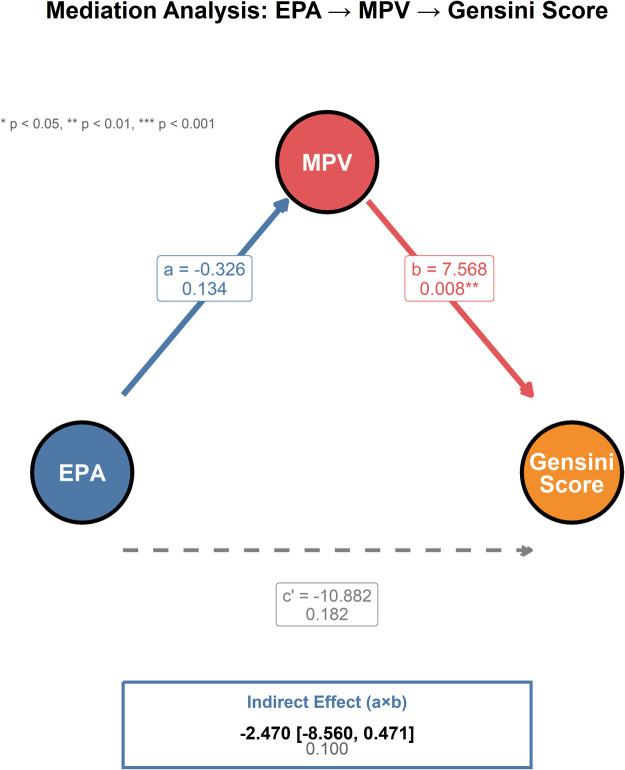
Analysis of the mediating effect of MPV on the relationship between EPA and gensini score. EPA, eicosapentaenoic acid; MPV, mean platelet volume.

In recent years, the cardiovascular protective effects of omega-3 polyunsaturated fatty acids have garnered significant attention. Research has confirmed that they can reduce the risk of CAD onset through mechanisms such as anti-inflammation, lipid regulation, and inhibition of platelet aggregation (8–11). However, differences in the biological activity and clinical benefit mechanisms between their core components, eicosapentaenoic acid (EPA) and docosahexaenoic acid (DHA), remain a current research focus. On one hand, research on EPA in the CAD field has achieved relatively clear progress. For instance, the RESPECT-EPA trial demonstrated that high-purity EPA can further reduce the risk of coronary events in patients with specific types of stable coronary heart disease (12). On the other hand, the protective effects of DHA are gaining increasing attention. Basic research by Bouhadoun et al. confirmed that DHA and its active metabolites RvD1, RvD5, and MaR1 can directly antagonize prostaglandin E-induced human coronary artery constriction, thereby structurally delaying the progression of atherosclerotic stenosis (13). Furthermore, a meta-analysis encompassing 13 studies revealed the most significant association between circulating DHA levels and reduced risk of heart failure (14). However, the distinct roles of EPA and DHA in the development of coronary heart disease (CHD) among the Chinese population and their underlying mechanisms remain unclear. Furthermore, given that platelet activation is a critical node in the pathophysiology of CAD and a key target for omega-3 fatty acids, whether the cardiovascular protective effects of omega-3 are partially mediated through inhibiting platelet activation and downregulating MPV warrants further investigation.

Therefore, this study aims to systematically examine the associations between *ω*-3 polyunsaturated fatty acids (*ω*-3 PUFAs, primarily EPA and DHA), MPV, and coronary artery stenosis severity and major adverse cardiovascular events (MACE) outcomes, and clarify the key mediating role of MPV in this process. This research seeks to offer novel theoretical basis and clinical translational evidence for the precise prevention and treatment of CAD.

## 2 Materials and methods

### 2.1 Study population

This study was conducted in compliance with the Declaration of Helsinki and was approved by the Ethics Committee of Foshan Second People's Hospital (No.: (2022)-0066) and the Eighth Affiliated Hospital of Southern Medical University (NO: KY20191103). Written informed consent was obtained from all participants. All participants were recruited from the two participating hospitals.

CAD was defined as ≥50% stenosis in at least one major coronary artery including the left main coronary artery, left anterior descending artery, left circumflex artery, and right coronary artery. Coronary artery stenosis was assessed and quantified by two independent interventional cardiologists using coronary angiography (CAG) or coronary computed tomography angiography (CCTA), with additional review by a radiologist from the participating hospital. Patients hospitalized in the cardiology department without a diagnosis of CAD were screened and enrolled as controls.

Patients were excluded if they had severe heart failure, cardiogenic shock, or other significant cardiac conditions, uncontrolled infectious diseases, autoimmune disorders, end-stage renal disease, psychiatric disorders, or malignancies,or if they had used fish oil or polyunsaturated fatty acid supplements in the preceding 3 months.

Follow-up for the CAD group lasted up to 52 months and concluded on December 31, 2024. Patients were enrolled from August 2020 to May 2023. The median follow-up duration was 38 months (IQR: 28–47 months), with a range of 19–52 months. The follow-up endpoint was the first occurrence of MACE (defined as cardiac death, non-fatal myocardial infarction, target vessel revascularization, or stroke) or the final follow-up visit. No patients were lost to follow-up. All 183 CAD patients completed the full follow-up period. During the follow-up period, 123 patients (67.2%) experienced at least one major adverse cardiovascular event. For outcome adjudication, MACE events were independently assessed by two cardiologists who were blinded to the patients’ fatty acid and MPV levels.

### 2.2 Laboratory parameter assessment

Platelet count (PLT), MPV, platelet distribution width (PDW), platelet large cell ratio (PLCR), hemoglobin (HGB), fasting blood glucose (FBG), glycated hemoglobin (HbA1c), total cholesterol (TC), high-density lipoprotein cholesterol (HDL-C), triglycerides (TG), low-density lipoprotein cholesterol (LDL-C), and serum creatinine (Scr) were measured in the laboratories of the participating hospitals and extracted from the medical records.

Traditional risk factors for coronary artery disease (CAD) used as covariates in this study were defined as follows: (1) Hypertension was defined in accordance with the current Chinese Hypertension Management Guidelines (15), including systolic blood pressure ≥140 mmHg and/or diastolic blood pressure ≥90 mmHg, or patients receiving antihypertensive therapy. (2) Type 2 diabetes mellitus was defined as FBG ≥7.0 mmol/L, HbA1c ≥ 6.5%, or patients receiving antidiabetic medication (16). (3) Dyslipidemia was defined as TC ≥ 5.2 mmol/L, LDL-C ≥ 3.4 mmol/L, HDL-C < 1.0 mmol/L, and/or TG ≥ 1.7 mmol/L, or a history of lipid-lowering therapy (17). (4) Regarding smoking status, smokers are defined as those who were still smoking within the 30 days prior to enrollment, non-smokers include those who have never smoked or who have quit smoking for ≥1 year.

### 2.3 Detections of eicosapentaenoic acid, docosahexaenoic acid, and arachidonic acid

Venous blood samples were collected after fasting for at least 8 h and stored at −80 °C for future measurement of free fatty acids. Serum levels of DHA, EPA, and AA were detected using ultra-high-performance liquid chromatography-mass spectrometry. In brief, the stored serum samples were thawed at 4 °C, and 50 mg of the samples were homogenized with 100 µL distilled water. After the addition of 0.5 mL of methanol, samples were extracted by vortexing for 30 min. After centrifuging at 14,000 rpm at 4 °C for 5 min, the supernatant was added with 5 µL of the inter-standard solution (FA19:0 25 µg/mL, diluted with methanol), then vortexed for 10 s, stored in a 2 mL injection vial for the test. After that, the UPLC analysis was performed using a Waters ACQUITY I-class LC system (Waters, Milford, MA, USA). Chromatographic separation was conducted on a Waters ACQUITY UPLC BEH C18 column (1.7 µm particle size, 2.1 mm × 100 mm), maintained at 55◦C. The mobile phase consisted of solvent A (Acetonitrile: water, 1:10, 1 mmol/L ammonium acetate) and solvent B (Isopropanol: Acetonitrile, 1:1). Gradient elution was carried out at a flow rate of 0.30 mL/min, with the injection volume of 1 µL. Mass spectrometry was performed using a Xevo TQ-S micro spectrometer (Waters, Milford, MA, USA).The following negative ion ESI parameters were used: turbo spray temperature 150 °C, spray voltage −2.5 kV, cone voltage 21 V, desolvation temperature 500 °C, and desolvation gas flow 1,000 L/h.

### 2.4 Statistical analysis

Categorical variables were presented as numbers and percentages. Continuous variables were first assessed for normality using the Shapiro–Wilk test and expressed as mean ± standard deviation (SD) or median and interquartile range (IQR). The Wilcoxon signed-rank test was used for non-normally distributed continuous variables, while the two-tailed *t*-test and the chi-square test with Yates continuity correction were applied to normally distributed continuous variables to compare baseline characteristics between CAD patients and the control group.

Univariate and multivariate logistic regression analyses were performed to identify factors associated with CAD. In the univariate analysis, potential associated variables were initially screened. Multivariate logistic regression was then used to further adjust for confounding factors, including age, sex, smoking status, hypertension, FBG, HbA1c, TC, TG, and low-density lipoprotein cholesterol. In the analysis, non-normally distributed continuous variables were subjected to log transformation to meet the model assumptions. Spearman's correlation coefficient (*ρ*) was used to assess the associations between *ω*-3 PUFAs and platelet morphological parameters (MPV, PDW, PLCR), as well as between *ω*-3 PUFAs, platelet parameters, and Gensini scores. The bootstrap method (1,000 simulations) was employed to validate the mediating effect of MPV on the relationship between fatty acids and Gensini scores. The indirect effect (ab) and its 95% confidence interval (95% CI) were calculated,a confidence interval excluding zero indicated a statistically significant mediating effect.

Survival curves for CAD patients were plotted using the Kaplan–Meier method, and intergroup differences were compared via the log-rank test. After adjusting for confounding factors including age, sex, hypertension, diabetes, TC, TG, HDL-C, and LDL-C in the Cox regression model, the independent predictive value of DHA and MPV was assessed.

All statistical analyses were performed using R software (version 4.5.1, R Foundation for Statistical Computing, Vienna, Austria). All *P*-values were two-tailed, and a *P* value <0.05 was considered statistically significant.

## 3 Results

### 3.1 Clinical characteristics of patients

In this study, 183 CAD patients (69.9% male) and 189 controls (50.8% male) were included for analysis. The baseline demographic and clinical characteristics of all participants are presented in Table 1.

**Table 1:** Demographic and clinical characteristics of CAD patients and controls.

Variables	All participants (*n* = 372)	Control group (*n* = 189)	CAD group (*n* = 183)	*P*-value
Age (years)	62.0 (51.00, 72.25)	65.0 (50.00, 74.00)	60.0 (51.50, 70.50)	0.334
Men (%)	224 (60.2%)	96 (50.8%)	128 (69.9%)	<0.001
Current smokers (*n*%)	130 (34.9%)	59 (31.2%)	71 (38.8%)	0.130
DM (*n*%)	120 (32.3%)	56 (29.6%)	64 (35.0%)	0.318
Hypertension (%)	238 (64.0%)	111 (58.7%)	127 (69.4%)	0.040
BMI (kg/m^2^)	24.0 (21.96, 26.19)	23.99 (21.84, 25.95)	24.04 (22.10, 26.45)	0.787
EPA (µmol/L)	0.37 (0.26, 0.55)	0.37 (0.21, 0.56)	0.36 (0.28, 0.55)	0.372
DHA (µmol/L)	1.71 (1.10, 2.91)	1.94 (1.24, 3.07)	1.41 (1.01, 2.56)	0.001
AA (µmol/L)	5.05 (3.79, 6.85)	5.29 (3.91, 6.84)	4.78 (3.51, 6.86)	0.242
PLT (×10^9^/L)	229.0 (186.75, 276.0)	224.0 (177.0, 268.0)	236.0 (194.0, 283.50)	0.039
MPV (fL)	10.30 (9.60, 10.90)	10.30 (9.70, 10.90)	10.40 (9.60, 10.95)	0.566
PDW (%)	11.60 (10.30, 12.90)	11.40 (10.30, 12.80)	11.80 (10.35, 12.95)	0.306
PLCR	0.27 (0.22, 0.32)	0.27 (0.22, 0.32)	0.28 (0.22, 0.33)	0.481
HGB (g/L)	132.00 (118.0, 145.0)	130.0 (115.00, 145.00)	136.0 (120.0, 146.0)	0.082
FBG (mmol/L)	6.24 (5.17, 8.48)	5.99 (5.01, 7.62)	6.65 (5.40, 9.02)	0.005
HbA1c (%)	5.90 (5.50, 6.50)	5.80 (5.50, 6.20)	6.00 (5.60, 6.90)	<0.001
TC (mmol/L)	4.18 (3.62, 5.10)	4.21 (3.59, 5.06)	4.16 (3.62, 5.12)	0.838
TG (mmol/L)	1.33 (0.95, 1.94)	1.19 (0.89, 1.59)	1.51 (1.04, 2.17)	<0.001
LDL-C (mmol/L)	2.53 (2.01, 3.11)	2.53 (2.03, 2.94)	2.55 (1.95, 3.27)	0.378
HDL-C (mmol/L)	1.07 (0.89, 1.28)	1.12 (0.92, 1.30)	1.02 (0.86, 1.22)	0.026
Scr (μmol/L)	77.88 (65.76, 101.52)	75.45 (62.15, 100.50)	80.0 (69.06, 105.75)	0.015

Compared with the control group, CAD patients had significantly higher proportions of males and hypertensive individuals, as well as elevated levels of PLT, FBG, HbA1c, TG, and Scr. In contrast, DHA and HDL-C levels were significantly lower in the CAD group (all *P* < 0.05). There was no statistically significant difference in the traditional risk factors for other cardiovascular diseases between the two groups (Table 1).

### 3.2 Analysis of risk factors for coronary heart disease

Results from univariate logistic regression analysis (with non-normally distributed variables log-transformed) showed that male sex (OR = 2.255, *P* < 0.001), hypertension (OR = 1.594, *P* = 0.0326), elevated PLT (OR = 2.196, *P* = 0.0214), elevated FBG (OR = 2.026, *P* = 0.0080), elevated HbA1c (OR = 5.376, *P* = 0.0048), elevated TG (OR = 2.451, *P* < 0.001), and elevated Scr (OR = 2.123, *P* = 0.0051) were potential risk factors for CAD (all *P* < 0.05). Conversely, higher DHA levels (OR = 0.671, *P* = 0.0125) was a protective factor for CAD (*P* < 0.05). Smoking, diabetes mellitus, age, BMI, EPA levels, AA levels, MPV, PDW, PLCR, TC, LDL-C, HDL-C, HGB, and other variables showed no significant association with CAD (all *P* > 0.05) (Table 2).

**Table 2:** Univariate logistic regression analysis of risk factors for coronary heart disease.

Variables	OR	95% CI	*P*-value
Current smokers	1.397	0.911–2.143	0.1258
Hypertension	1.594	1.039–2.444	0.0326
DM	1.277	0.826–1.975	0.2708
Men	2.255	1.473–3.451	<0.001
Lnage	1.117	0.512–2.436	0.7814
LnBMI	1.354	0.340–5.398	0.6673
LnEPA	1.291	0.938–1.776	0.1166
LnDHA	0.671	0.490–0.917	0.0125
LnAA	0.899	0.586–1.381	0.6279
LnPLT	2.196	1.123–4.293	0.0214
LnMPV	1.620	0.182–14.418	0.6655
LnPDW	1.540	0.470–5.041	0.4756
LnPLCR	1.038	0.547–1.969	0.9103
LnFBG	2.026	1.202–3.416	0.0080
LnHbA1c	5.376	1.673–17.281	0.0048
LnTC	1.420	0.667–3.022	0.3631
LnLDLC	1.422	0.784–2.577	0.2462
LnHDLC	0.643	0.309–1.339	0.2382
LnTG	2.451	1.648–3.646	<0.001
LnHGB	2.301	0.730–7.251	0.1547
LnScr	2.123	1.253–3.595	0.0051

To further identify independent risk factors for CAD, variables with *P* < 0.1 in the univariate analysis (including male sex, hypertension, lnDHA, lnPLT, lnFBG, lnHbA1c, lnTG, and lnScr) were included in a multivariate logistic regression model. After adjusting for confounding factors, including age, sex, hypertension, smoking history, FBG, HbA1c,TC, TG, and low-density lipoprotein cholesterol, the multivariate logistic regression results revealed that male sex (OR = 2.585, 95% CI: 1.366–4.892, *P* = 0.0035), lnPLT (OR = 2.426, 95% CI: 1.115–5.281, *P* = 0.0255), and lnTG (OR = 2.203, 95% CI: 1.379–3.519, *P* = 0.0009) were independent risk factors for CAD (all *P* < 0.05). In contrast, lnDHA was an independent protective factor for CAD (OR = 0.704, 95% CI: 0.497–0.997, *P* = 0.0479) (Table 3).

**Table 3:** Multivariate logistic regression analysis of risk factors for coronary heart disease.

Variables	OR	95% CI	*P*-value
Current smokers	0.787	0.449–1.381	0.4042
HBP	1.313	0.783–2.203	0.3021
Men	2.585	1.366–4.892	0.0035
Lnage	2.032	0.706–5.848	0.1888
LnDHA	0.704	0.497–0.997	0.0479
LnPLT	2.426	1.115–5.281	0.0255
LnFBG	1.381	0.675–2.826	0.3762
LnHbA1c	1.164	0.228–5.926	0.8553
LnTC	1.075	0.197–5.881	0.9334
LnTG	2.203	1.379–3.519	0.0009
LnLDLC	1.115	0.310–4.013	0.8679
LnScr	1.322	0.722–2.422	0.3656

### 3.3 Correlation between fatty acids and platelet parameters

Table 4 presents the results of Spearman correlation analyses between fatty acids and platelet parameters, stratified by CAD status. No significant associations were observed in the control group. However, among patients with CAD, EPA levels were significantly negatively correlated with MPV (*r* = −0.165, *P* = 0.025) and PLCR (*r* = −0.178, *P* = 0.016). DHA levels were significantly negatively correlated with MPV (*r* = −0.169, *P* = 0.022), PDW (*r* = −0.156, *P* = 0.035), and PLCR (*r* = −0.186, *P* = 0.012). AA levels showed no statistically significant correlation with any platelet parameters in either group (all *P* > 0.05).

**Table 4:** Correlation between fatty acids and platelet parameters.

Fatty acids	Platelet parameters	Control group (*n* = 189)		CAD group (*n* = 183)	
		*R*-value	*P*-value	*R*-value	*P*-value
EPA	MPV	0.015	0.837	−0.165	0.025
	PDW	0.013	0.858	−0.144	0.052
	PLCR	0.019	0.795	−0.178	0.016
DHA	MPV	0.078	0.287	−0.169	0.022
	PDW	0.066	0.370	−0.156	0.035
	PLCR	0.078	0.285	−0.186	0.012
AA	MPV	0.009	0.900	−0.090	0.223
	PDW	0.015	0.835	−0.108	0.144
	PLCR	0.014	0.848	−0.125	0.093

### 3.4 Correlation between fatty acids, platelet parameters, and gensini score

Spearman correlation analysis revealed that in patients with CAD, MPV, PDW, and PLCR all showed positive correlations with Gensini scores (*r* values of 0.17, 0.158, and 0.165, respectively, all *P* < 0.05), with MPV exhibiting the strongest correlation (*r* = 0.17, *P* = 0.022). PLT and fatty acid markers (AA, EPA, DHA) showed no significant correlation with Gensini scores (all *P* > 0.05) (Table 5). Further analysis revealed extremely strong correlations among MPV, PDW, and PLCR (MPV with PDW: *r* = 0.931, MPV and PLCR: *r* = 0.976, PDW and PLCR: *r* = 0.969). Variance inflation factor (VIF) analysis confirmed VIF values of 12.44 for both PDW and PLCR, indicating severe multicollinearity. To mitigate multicollinearity interference in subsequent analyses and considering correlation strength, MPV was selected as the representative platelet morphological parameter for the mediation effect analysis.

**Table 5:** Correlation analysis between platelet parameters and gensini score.

Variables	*R*-value	*P*-value
MPV	0.17	0.022
PDW	0.158	0.032
PLCR	0.165	0.025
PLT	0.07	0.353
EPA	−0.017	0.814
DHA	−0.050	0.504
AA	−0.054	0.467

### 3.5 Analysis of the mediating effect of platelet parameters on the relationship between fatty acids and coronary artery stenosis severity

After adjusting for confounding factors including age, gender, hypertension, diabetes mellitus, smoking history, TC, HDL-C, and LDL-C, mediation analyses were performed using EPA and DHA as independent variables, the core platelet morphological parameter MPV as the mediator, and the Gensini score as the dependent variable (based on 183 CAD patients, with 1,000 repeated simulations using the bootstrap method). The results showed that DHA exerted a marginally significant negative effect on MPV (path coefficient *a* = –0.066, *P* = 0.069), whereas MPV significantly and positively influenced Gensini scores (path coefficient *b* = 7.352, *P* = 0.010). The direct effect of DHA on the Gensini score was not statistically significant (direct effect *c*′ = –2.239, *P* = 0.1), but its indirect effect mediated by MPV was significant [*a* × *b* = –0.486, 95% confidence interval: (–1.370, −0.021),interval does not contain 0, *P* = 0.034] (Figure 1). In contrast, the mediating effect of MPV on the relationship between EPA and the Gensini score was not statistically significant. These findings suggest that the association between DHA and lower Gensini scores may be statistically explained in part by MPV levels, whereas the corresponding effect of EPA was not reflected through this MPV-mediated pathway (Figure 2).

### 3.6 Multifactorial Cox proportional hazards regression model analysis of risk factors for MACE in patients with coronary artery disease

**Figure 3:**
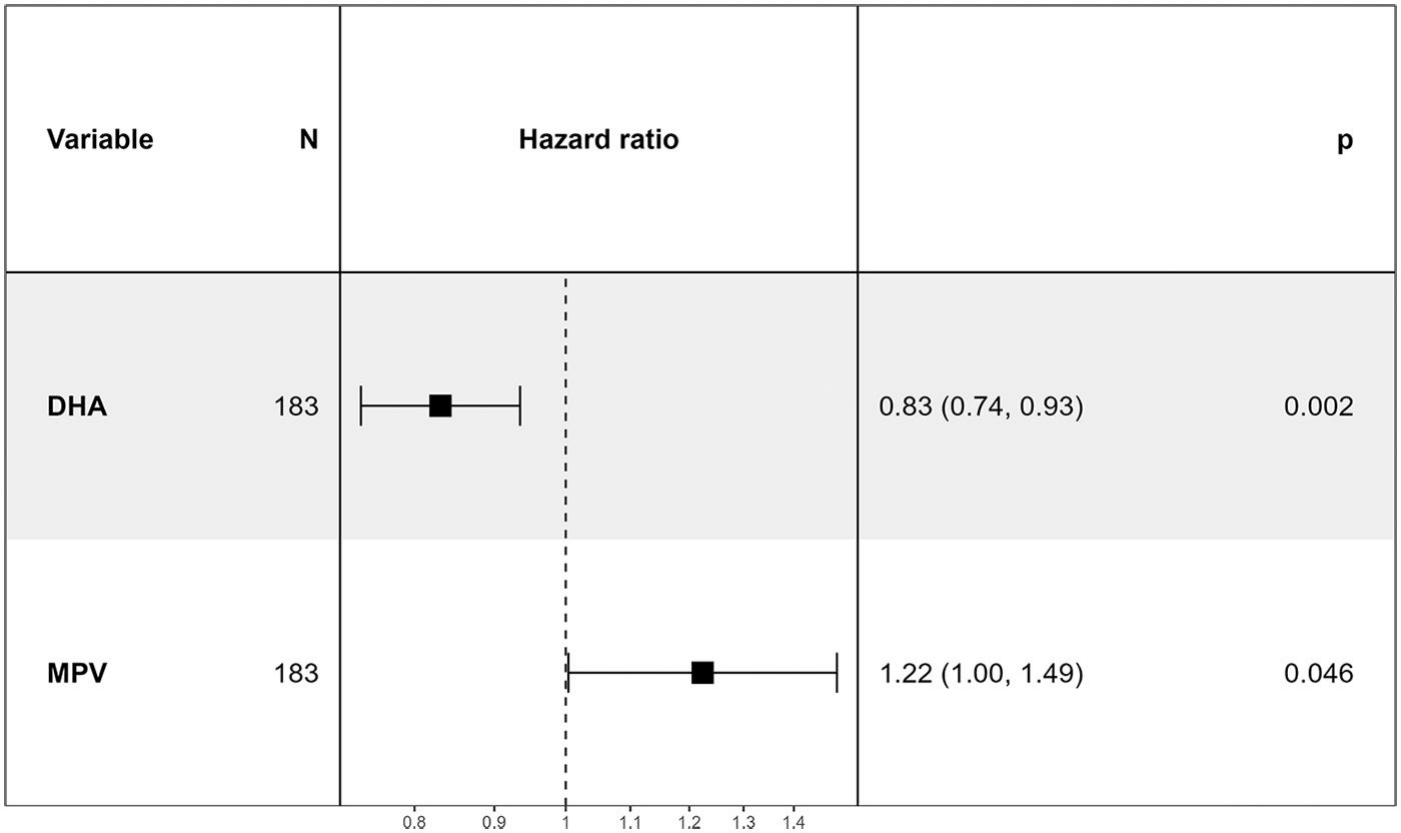
Multifactorial Cox proportional hazards regression model analysis of risk factors for Major adverse cardiovascular events in patients with coronary heart disease. DHA, docosahexaenoic acid; MPV, mean platelet volume.

Multivariate Cox proportional hazards regression analysis, encompassing 183 CAD patients, revealed that after adjusting for confounding factors including age, sex, hypertension, diabetes mellitus, TC, TC, HDL-C, and LDL-C, DHA emerged as an independent protective factor for MACE (HR = 0.83, 95% CI: 0.74–0.93, *P* = 0.002), indicating a 17% reduction in MACE risk per 1-unit increase in DHA level. MPV was identified as an independent risk factor for MACE (HR = 1.22, 95% CI: 1.00–1.49, *P* = 0.046), meaning that for every 1 fL increase in MPV, the risk of MACE increased by 22% (Figure 3).

### 3.7 The impact of fatty acids and platelet parameters on the prognosis of MACE in patients with CAD

**Figure 4:**
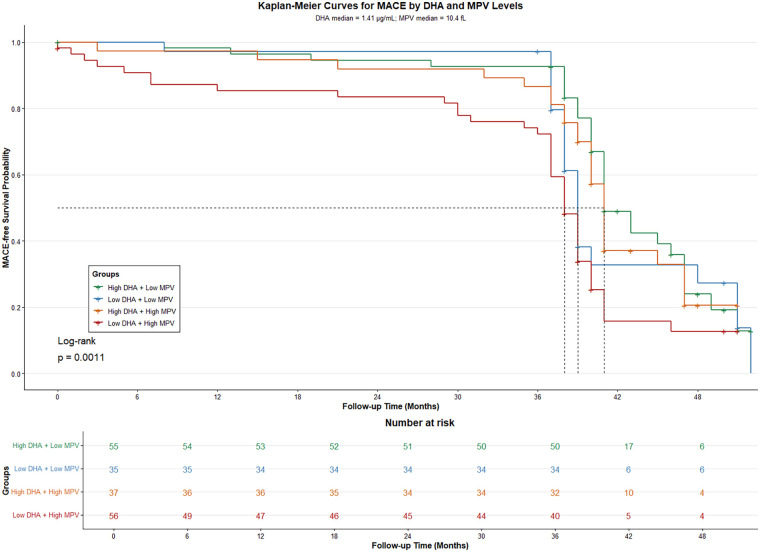
Kaplan–Meier curves for mace by DHA and MPV levels. DHA, docosahexaenoic acid; MPV, mean platelet volume.

Kaplan–Meier curve analysis (Figure 4) revealed that when patients with CAD were grouped according to the combined median levels of DHA (1.4127 µmol/L) and MPV (10.4 fL), there were statistically significant differences in MACE-free survival among the four groups (*P* = 0.0011). The “high DHA + low MPV” group consistently maintained the lowest MACE-free survival rate. Pairwise comparison results (Table 6) showed that compared with the “high DHA + low MPV” group, the “low DHA + high MPV” group had a 2.35-fold increased risk of MACE (HR = 2.35, 95% CI: 1.47–3.75, *P* < 0.001).

**Table 6:** Comparisons of MACE-free survival.

Comparison	Group 1 MACE% (n/N)	Group 2 MACE% (n/N)	HR (95% CI)	*P*-value	*P*-adjusted*
High DHA + Low MPV vs. Low DHA + High MPV	60.0% (33/55)	73.2% (41/56)	2.35 (1.47–3.75)	<0.001	0.0012
High DHA + High MPV vs. Low DHA + High MPV	67.6% (25/37)	73.2% (41/56)	1.95 (1.17–3.24)	0.0075	0.0450
Low DHA + Low MPV vs. Low DHA + High MPV	68.6% (24/35)	73.2% (41/56)	1.42 (0.85–2.37)	0.1321	0.7926
High DHA + Low MPV vs. Low DHA + Low MPV	60.0% (33/55)	68.6% (24/35)	1.59 (0.92–2.73)	0.0942	0.5652
Low DHA + Low MPV vs. High DHA + High MPV	68.6% (24/35)	67.6% (25/37)	0.75 (0.42–1.33)	0.3354	1.0000
High DHA + Low MPV vs. High DHA + High MPV	60.0% (33/55)	67.6% (25/37)	1.16 (0.69–1.95)	0.5963	1.0000

*P*-adjusted*: *P* values were adjusted for multiple pairwise comparisons using the Bonferroni method.

## 4 Discussion

This study found that in Chinese patients with coronary artery disease (CAD), docosahexaenoic acid (DHA) can influence the degree of coronary artery stenosis by mediating platelet morphological parameters centered on MPV. The combination of “high DHA + low MPV” significantly reduces the risk of MACE, providing a novel combined risk stratification indicator for CAD patients.

The anti-atherosclerotic effects of *ω*-3 PUFAs have been well established. Previous studies have primarily focused on their direct association with coronary stenosis (18, 19), without clarifying the specific effect indicators or mediating pathways underlying “*ω*-3 PUFAs inhibiting platelet aggregation.” This study identifies MPV as a key mediator between DHA and coronary artery stenosis severity in CAD patients. Omega-3 PUFAs are essential structural components of the phospholipid bilayer in cell membranes. Research indicates that EPA and DHA are incorporated into platelet phospholipids at the expense of arachidonic acid (AA), thereby reducing the synthesis of the AA-derived metabolite thromboxane A2 (TXA2) and inhibiting platelet aggregation (20). A meta-analysis by Gao et al. confirmed that supplementation with polyunsaturated fatty acids (primarily DHA/EPA, 0.85–6.8 g/day) reduced ADP-induced platelet aggregation by 23% (*P* = 0.02) (21). Moertl et al. confirmed that long-chain omega-3 polyunsaturated fatty acids (LC*ω*-3 PUFAs) can dose-dependently reduce platelet activation and tissue factor levels in patients with chronic heart failure (22), further suggesting that the regulation of platelet function by *ω*-3 PUFAs is a crucial component of their cardiovascular protective effects. Furthermore, Woodman et al. found that DHA significantly reduced platelet aggregation and thromboxane B2 levels in patients with hypertension and type 2 diabetes, supporting the notion that high-purity DHA may exert stronger antithrombotic effects than EPA (23). This aligns with our finding that DHA (rather than EPA) mediates its effects via MPV, indirectly suggesting that DHA may possess more potent and targeted regulation of platelet function.

Cox proportional hazards regression analysis confirmed that both DHA and MPV are independent predictors of MACE risk in this population. Building on these findings, Kaplan–Meier survival curve analysis demonstrated that the combined “high DHA + low MPV” assessment confers superior prognostic stratification value for CAD patients.

On the one hand, elevated DHA levels exert protective effects by inhibiting platelet activation and reducing thrombogenesis (24). The present study identified DHA as a significant protective factor against CAD, which is consistent with the findings of a recent large-scale Mendelian randomization study. This study genetically confirmed that higher circulating DHA levels are a protective factor against CAD-related mortality risk (25). Similarly, Harris et al. validated through 17 prospective cohort studies that elevated DHA levels are significantly associated with a lower risk of cardiovascular death (26). The MERLIN-TIMI 36 study further demonstrated that high plasma long-chain omega-3 PUFAs reduce the risk of sudden cardiac death by 63% in patients with non-ST-segment elevation acute coronary syndrome (ACS) (27). On the other hand, elevated MPV reflects increased platelet volume and enhanced reactivity,the dense granules in these platelets release higher levels of proinflammatory and prothrombotic substances (e.g., thromboxane A2 and P-selectin), thereby exacerbating plaque instability and thrombosis (28). A 2024 systematic review further confirmed that elevated MPV is an independent predictor of MACE in patients with CAD (29), a finding that is equally valid in acute ischemic stroke (30), supporting MPV's universality of MPV as a core biomarker for arterial thrombotic diseases.

The combined assessment of DHA and MPV provides a novel and more comprehensive dimension for clinical risk stratification in CAD patients. For high-risk individuals with the “low DHA + high MPV” profile, DHA supplementation should be prioritized alongside enhanced platelet function monitoring to reduce MACE risk.

This study has several limitations. First, the mediation analysis can only reveal an association and cannot directly establish a causal relationship between “DHA, MPV, and coronary stenosis”, the proposed pathway remains a mechanistic hypothesis that requires further validation through prospective intervention trials. Second, this study included only 183 CAD patients, representing a relatively small sample size. Additionally, coronary lesions were assessed using CTA in some patients, and the study did not collect or adjust for the use of antiplatelet drugs (aspirin, clopidogrel, ticagrelor) or statins. These medications may influence platelet function, MPV levels, and MACE risk, constituting important unmeasured confounding factors that could compromise the accuracy of the results. Third, all study participants were from southern Guangdong Province, China, due to regional dietary differences, the generalizability of the conclusions is limited. Fourth, the use of conventional platelet parameters such as MPV to assess platelet activity lacks precision compared to specific indicators like platelet aggregation rate and thromboxane B_2_, and the study did not explore the interactions between DHA and other fatty acids.

To address these limitations, future research could be deepened in the following directions: First, conduct multicenter, large-sample prospective cohort studies that include populations from different regions, age groups, and with varying underlying medical conditions to systematically validate the causal relationship and generalizability of the “*ω*-3 fatty acids—MPV—coronary stenosis” pathway,second, design randomized controlled trials (RCTs) with graduated-dose *ω*-3 fatty acid intervention groups to dynamically monitor changes in platelet parameters, platelet function indices, and the degree of coronary stenosis, thereby clarifying causal effects and refining dose–response relationships at the intervention level, Third, we will expand mediating and moderating effect models by incorporating potential variables such as inflammatory factors and coagulation function indicators to explore multi-pathway synergistic regulatory mechanisms, while simultaneously analyzing interactions among fatty acids, fourth, we will integrate genomics or metabolomics technologies to screen for key genetic loci or metabolic biomarkers influencing the pathway, thereby providing refined theoretical support for precision prevention and treatment.

## 5 Conclusion

DHA can influence the severity of coronary artery stenosis in patients with CAD by mediating platelet parameters centered on MPV. Combining DHA supplementation with MPV testing optimizes risk stratification in patients with CAD, offering quantitative evidence for personalized nutritional interventions with significant clinical translational value.

## Data Availability

The original contributions presented in the study are included in the article/Supplementary Material, further inquiries can be directed to the corresponding author.
